# Characterization of Protocatechuate 4,5-Dioxygenase from *Pseudarthrobacter phenanthrenivorans* Sphe3 and In Situ Reaction Monitoring in the NMR Tube

**DOI:** 10.3390/ijms22179647

**Published:** 2021-09-06

**Authors:** Epameinondas Tsagogiannis, Elpiniki Vandera, Alexandra Primikyri, Stamatia Asimakoula, Andreas G. Tzakos, Ioannis P. Gerothanassis, Anna-Irini Koukkou

**Affiliations:** 1 Laboratory of Biochemistry, Sector of Organic Chemistry and Biochemistry, Department of Chemistry, University of Ioannina, 45110 Ioannina, Greece; e.tsagkogiannis@uoi.gr (E.T.); evandera@uoi.gr (E.V.); s.asimakoula@uoi.gr (S.A.); 2 Laboratory of Organic Chemistry, Sector of Organic Chemistry and Biochemistry, Department of Chemistry, University of Ioannina, 45110 Ioannina, Greece; a.primikyri@uoi.gr (A.P.); atzakos@uoi.gr (A.G.T.); igeroth@uoi.gr (I.P.G.)

**Keywords:** *Pseudarthrobacter phenanthrenivorans* Sphe3, protocatechuate (PCA) 4,5-dioxygenase, in situ biotransformation monitoring, NMR bioreactor, extradiol dioxygenases, Actinobacteria

## Abstract

The current study aims at the functional and kinetic characterization of protocatechuate (PCA) 4,5-dioxygenase (PcaA) from *Pseudarthrobacter phenanthrenivorans* Sphe3. This is the first single subunit Type II dioxygenase characterized in Actinobacteria. RT-PCR analysis demonstrated that *pca*A and the adjacent putative genes implicated in the PCA *meta*-cleavage pathway comprise a single transcriptional unit. The recombinant PcaA is highly specific for PCA and exhibits Michaelis–Menten kinetics with K_m_ and V_max_ values of 21 ± 1.6 μM and 44.8 ± 4.0 U × mg^−1^, respectively, in pH 9.5 and at 20 °C. PcaA also converted gallate from a broad range of substrates tested. The enzymatic reaction products were identified and characterized, for the first time, through in situ biotransformation monitoring inside an NMR tube. The PCA reaction product demonstrated a keto-enol tautomerization, whereas the gallate reaction product was present only in the keto form. Moreover, the transcriptional levels of *pca*A and *pca*R (gene encoding a LysR-type regulator of the pathway) were also determined, showing an induction when cells were grown on PCA and phenanthrene. Studying key enzymes in biodegradation pathways is significant for bioremediation and for efficient biocatalysts development.

## 1. Introduction

Protocatechuic acid (PCA) is an important ring-cleavage common intermediate formed from the catabolism of low molecular weight polycyclic aromatic hydrocarbons, namely ferulic, vanillic, and *p*-coumaric acid. These compounds are derived from the apopolymerization of lignin, phthalic acid isomers, chlorobenzoic acids, and methylated aromatic hydrocarbons such as toluene and xylene [1].

PCA is further metabolized and funneled to the TCA cycle under aerobic conditions by the action of intradiol or extradiol dioxygenases via three distinct catabolic pathways, differing in the location of the initial ring opening oxidation: 2,3-dioxygenation (*para*-cleavage pathway) [2,3], 3,4-dioxygenation (*ortho*-cleavage, known as the *β*-ketoadipate pathway) [4], and 4,5-dioxygenation of PCA (*meta*-cleavage pathway) [5]. Generally, the *ortho*-cleavage pathway of PCA is widely distributed amongst Actinobacteria and Proteobacteria [6] and PCA 3,4-dioxygenase is the most commonly characterized enzyme [7].

In contrast, homologues of PCA 4,5-dioxygenases (*meta*-cleavage pathway) were found almost exclusively in Proteobacteria [8,9], whereas Actinobacteria degrade PCA predominantly via the *ortho*-cleavage pathway [9]. Perez-Pantoja and co-workers [10] reported that only the genome of *Arthrobacte*r sp. FB24 among the 53 actinobacterial genomes, analyzed in a phylogenetic study, possesses a PCA 4,5-dioxygenase gene. The *meta*-cleavage enzymes have been studied less relative to the respective *ortho*-cleavage enzymes, probably due to their sparser distribution among bacteria and/or their relative instability [11].

The hetero-multimeric PCA 4,5-dioxygenases, composed of two subunits with the encoding genes adjacently located, belong to the PCA dioxygenase (PCAD) of the phosphorylase/peptidyl hydrolase fold (Type II) of extradiol-bond cleaving dioxygenases [12,13,14]. PCAD superfamily domains exhibit structural and sequence similarities with the Memo-protein superfamily that led Burroughs et al. [14] to their classification as the novel PCAD-Memo superfamily.

Although numerous biochemical characterizations of the PCA 4,5-dioxygenases have been established in several bacterial groups such as *Comamonas* species [5,8,15,16], *Pseudomonas* species [17], *Sphingomonas* species [12,18,19,20], *Delftia* sp. strain TBKNP-05 [21], and *Rhizobium leguminosarum* [11], until now, the PCA 4,5-dioxygenase (LigAB) from *Sphingomonas paucimobilis* SYK-6 is the only structurally and fully kinetically characterized enzyme [12].

*Pseudarthrobacter phenanthrenivorans* Sphe3 (formerly *Arthrobacter phenanthrenivorans* Sphe3) [22] catabolizes phenanthrene at concentrations up to 400 mg/Las a sole source of carbon and energy at higher rates than those reported for any other member of the genus [23,24]. In silico analysis of the Sphe3 genome [25] revealed putative gene clusters on pASPHE302 plasmid and the chromosome, implicated in the *meta*- and *ortho*-cleavage of PCA, respectively, in contrast to other members of the genus *Arthrobacter* that catabolize PCA only via the *ortho*-cleavage pathway [26,27,28]. The presence of both *ortho-* and *meta*-cleavage pathways in Sphe3 was also validated through proteomic analysis [29]. Although the occurrence of *meta*-cleavage of PCA has also been demonstrated in *Arthrobacter keyseri* 12B [30] as well as in other Actinobacteria [31], there is no study on the kinetic characterization of PCA 4,5- dioxygenase in this phylum. The functional and/or kinetic studies of enzymes belonging to the largely uncharacterized Type II extradiol dioxygenase clade of the PCAD-Memo superfamily will allow comparisons among these enzymes, thus shedding more light on this Type in the dioxygenase superfamily.

In the present study, we report the heterologous expression in *E. coli* and biochemical characterization of the recombinant PCA 4,5-dioxygenase (PcaA). Substrate specificity of the enzyme was also assessed and NMR spectroscopy was employed for the in situ monitoring of PCA biotransformation by PcaA and the identification of its products. To our knowledge, this is the first report on the biochemical characterization of the PCA 4,5-dioxygenase from the genus *Arthrobacter* and recording in real-time the formation of biotransformation products using a 5 mm NMR tube bioreactor.

Furthermore, the co-transcription of the genes involved in the PCA 4,5-cleavage pathway was demonstrated using RT-PCR, and the transcriptional levels of the genes encoding the PCA 4,5-dioxygenase and a LysR-type regulator of the 4,5-cleavage pathway were analyzed by quantitative real-time PCR (RT-qPCR) in cells grown in the presence of phenanthrene and protocatechuic acid.

## 2. Results

### 2.1. PCA 4,5-Dioxygenase Activity and Transcriptional Analysis with RT-PCR and RT-qPCR

**Figure 1:**
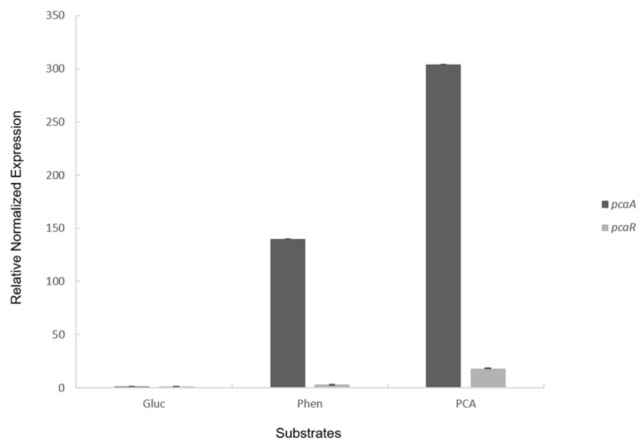
*pca*A and *pca*R gene expression quantification monitored by RT-qPCR in *P. phenanthrenivorans* Sphe3 grown on glucose, phenanthrene, and PCA as the sole carbon and energy source. Values represent the mean relative *pca*A and *pca*R expression normalized to the housekeeping gene *gyr*β ± standard deviations of three individual replicates. Gene expression levels in glucose were used as calibrator.

The activity of PcaA was measured in a crude extract of mid logarithmic phase Sphe3 cells grown on phenanthrene, PCA, or glucose. Higher activity was recorded in cells grown on PCA (0.025 U × mg^−1^) than on phenanthrene (0.012 U × mg^−1^) whereas no activity was detected in cells grown on glucose. To confirm the transcriptional induction of the PcaA, its expression was monitored by RT-qPCR in cultures of Sphe3 on M9 with glucose, phenanthrene, or PCA as the sole carbon source. The expression of *pca*A gene was induced in cells grown on phenanthrene or PCA showing up to 139- and 304-fold change increase in mRNA expression levels in the presence of phenanthrene and PCA, respectively, when compared to glucose (Figure 1).

**Figure 2:**
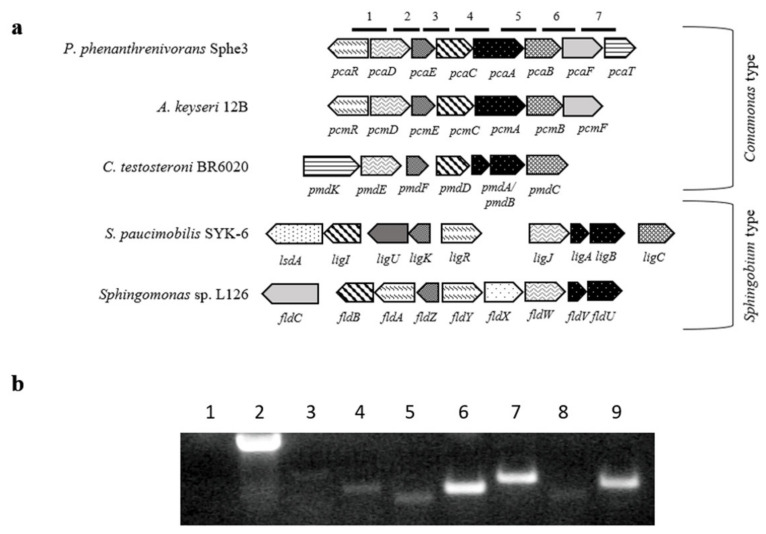
(**a**) *Sphingobium* and *Comamonas* types of genetic organization of gene clusters from the PCA 4,5-dioxygenation pathway in various microorganisms [16,19,20,30]. Genes: *pca*R (Asphe3_42340), *pcm*R, *fld*Y, putative transcriptional regulatory gene; *lig*R, transcriptional regulatory gene; *pca*D (Asphe3_42350), *pcm*D, *pmd*E, *lig*J, *fld*W, OMA hydratase; *pca*E (Asphe3_42360), *pcm*E, *pmd*F, *lig*K, *fld*Z, CHA aldolase; *pca*C (Asphe3_ 42370), *pcm*C, *pmd*D, *lig*I, *fld*B, PDC hydrolase; *pca*A (Asphe3_42380), *pcm*A, PCA 4,5-dioxygenase; *pmd*A, *lig*A, *fld*V, *α*-subunit of PCA 4,5-dioxygenase; *pmd*B, *lig*B, *fld*U, *β*-subunit of PCA 4,5-dioxygenase; *pca*B (Asphe_42390), *pcm*B, *pmd*C, *lig*C, CHMS dehydrogenase; *pcm*F, *fld*C, putative alcohol dehydrogenase; *pca*G (Asphe_42400), putative oxidoreductase, aryl-alcohol dehydrogenase like protein; *pca*T (Asphe3_42410), ABC-type molybdate transport system; *lsd*A, putative lignostilbene *α*,*β*-dioxygenase; *lig*U, *fld*A, 4-oxalomesaconate tautomerase. Numbered lines on the top part of the image indicate the regions according to which primers were designed in order study whether the genes of PCA 4,5-dioxygenation pathway are part of the same transcription unit, with reverse transcription-PCR, as follows: 1. pcaRT1, 2. pcaRT2, 3. pcaRT3, 4. pcaRT4, 5. pcaRT5, 6. pcaRT6, pcaRT7 (also see Table S1). (**b**) Agarose gel electrophoresis of co-transcription products of RT-PCR. Lane 1. no DNA control, 2. positive DNA control, 3. pcaRT1 product, 4. pcaRT2 product, 5. pcaRT3 product, 6. pcaRT4 product, 7. pcaRT5 product, 8. pcaRT6 product, 9. pcaRT7 product.

In strain Sphe3, a *pca*R gene encoding a LysR-type transcriptional regulatory protein was identified upstream of the respective operon and inversely oriented to the rest of the genes (Figure 2a). The expression of *pca*R was induced during growth on phenanthrene or PCA. However, higher expression of *pca*R on PCA (18-fold) than on phenanthrene (3-fold) was observed, whereas a minimal transcription of *pca*R was detected in cultures grown in the presence of glucose. These results indicate a regulation but with a basal constitutive expression of *pca*R gene (Figure 1). Transcriptional analysis with RT-PCR resulted in amplification products of the predicted sizes in all cases (Figure 2b), suggesting that PCA 4,5-pathway genes are transcribed as a single transcriptional unit.

In strain Sphe3, a single subunit which contains the regions corresponding to the *α* and *β* subunit of PCA 4,5-dioxygenases at the N- and C-terminals, respectively, was identified by in silico analysis of the genome, something that was also previously reported for strain *A. keyseri* 12B [30].

**Figure 3:**
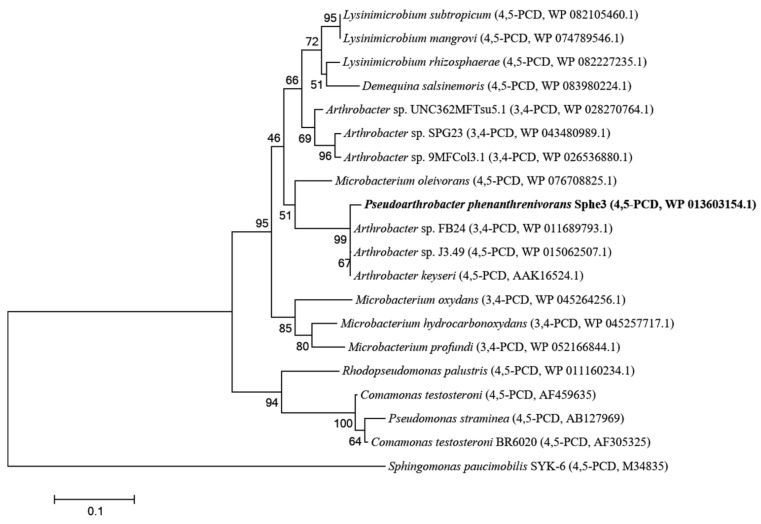
Phylogenetic tree of PcaA and other PCA dioxygenases. The dioxygenases used for the construction of the phylogenetic tree presented >66% identity with PcaA. The tree was constructed with the neighbor joining algorithm. The percentage of replicate trees in which the associated dioxygenases clustered together in the bootstrap test (500 replicates) is shown next to the branches. The scale bar indicates the number of substitutions per nucleotide position. Accession numbers are in parentheses. 3,4-PCD: PCA 3,4-dioxygenase; 4,5-PCD: PCA 4,5-dioxygenase.

The predicted protein product of the PCA 4,5-dioxygenase gene shared 99% sequence identity with the PCA 4,5-dioxygenase from *A. keyseri* 12B [30] at the amino acid level. The catalytic *β* subunit of the enzyme also shares 67% and 66% amino acid sequence identity with the *β* subunits of PCA 4,5-dioxygenases from *C. testosteroni* [15] and *P. straminea* [17], respectively. Multiple sequence alignment of PcaA with other extradiol dioxygenases using CLUSTALW algorithm revealed key conserved features of the enzyme (Figure S1). Phylogenetic and molecular evolutionary analysis of PcaA using amino acid sequences with MEGA algorithm, showed that *P. phenanthrenivorans* is in the same sister taxon of *Arthrobacter* sp. FB24, *Arthrobacter* sp. J3.49, and *A. keyseri* 12B (Figure 3).

### 2.2. Heterologous Expression and Catalytic Properties of the Recombinant PcaA

The recombinant PcaA resulted in a protein band of ~45 kDa, as determined by SDS-PAGE analysis (Figure S2), corresponding well to the in silico predicted MW of 48 kDa. The recombinant enzyme was active within pH 5.5 and 10.5, with an optimum at pH 9.5, when spectrophotometrically determined, while at pH 5.0 and pH 11.0, the enzyme completely lost its activity. The enzymatic reaction product, 4-carboxy-2-hydroxymuconate-6-semialdehyde (CHMS), exhibits a yellow color with an absorption maximum in alkaline solution (pH 9.0) [32]. In order to examine if the higher activity is due to the higher absorbance in pH 9.5, PcaA activity was recorded in the same pH range by O_2_ consumption rate. These measurements were also consistent with the spectrophotometric determination further verifying that the optimum pH for PcaA is 9.5. The effect of temperature on PcaA activity was evaluated in 100 mM Glycine-NaOH buffer (pH 9.5) at temperatures ranging between 5 °C and 50 °C. The enzyme was found to be active between 10 °C and 45 °C with an optimum activity at 20 °C. Its activity was remarkably reduced to 20% relative activity at 10 °C and 45 °C.

The recombinant PcaA exhibited PCA 4,5-dioxygenase activity estimated at 6.18 U × mg^−1^, at the optima pH 9.5 and 20 °C. After incubation of the enzyme with 0.2 mM Fe (II) and L-ascorbic acid, a significant increase, more than a 6-fold, in specific activity was observed (39.65 U × mg^−1^). The apparent *K_m_* value of PcaA for PCA as estimated at 20 °C was 21 ± 1.6 μM and the V_max_ was found 44.8 ± 4.0 U × mg^−1^.

Incubation of PcaA with metal-chelating agents such as bipy, EDTA or *o*-phenanthroline, was insufficient to significantly decrease the PcaA activity (data not shown). On the other hand, incubation with various concentrations of Cu(II) and Mn(II) exhibited strong inhibitory effects (the enzyme lost 87% and 57% of its relative activity, respectively), unlike Mg(II) and Ni(II), which caused no significant inhibition (less than 10% inhibition in both cases).

The purified enzyme maintained 93% of its activity for up to at least 4 months upon storage at −80 °C in the presence of 10% (*v*/*v*) glycerol. High levels of activity maintenance were recorded in the presence of 1% (*v*/*v*) DMSO, while ethanol (10% (*v*/*v*)) or higher DMSO concentrations (10% (*v*/*v*)) failed to maintain high levels of activity.

### 2.3. Substrate Specificity

Of the potential substrates that were evaluated, only gallate (5-hydroxyprotocatechuate) exhibited oxygen consumption activity, corresponding to 44% of the enzyme’s activity. No activity was recorded with homoprotocatechuate (3,4-dihydroxyphenylacetate) or any of the other substrates tested that contain the catechol scaffold.

### 2.4. In Situ Monitoring of the PcaA Enzymatic Reaction Using PCA or Gallate as Substrates in the NMR Tube Bioreactor

**Figure 4:**
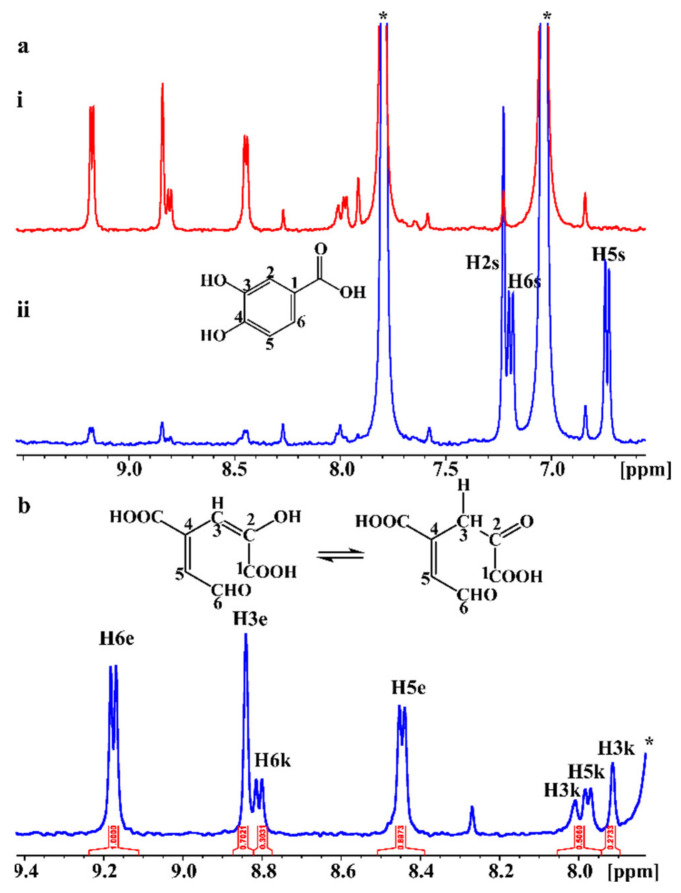
In situ monitoring of the biotransformation of PCA by PcaA, inside the NMR tube. (**a**) ^1^H NMR spectra 2 h (blue spectrum, (**i**)) and 24 h (red spectrum, (**ii**)) after the addition of PCA to PCA 4,5 dioxygenase. ^1^H NMR resonances of the substrate are shown in the blue spectrum. The asterisk (*) denotes protons of imidazole present in the buffer. (**b**) Expanded region (7.8–9.4 ppm) of the ^1^H NMR spectrum of Figure 4aii and the chemical structures of the enol (left) and keto (right) forms (number of scans = 64, acquisition time = 4 s, relaxation delay = 1.5 s, total experimental time = 6 min 22 s).

The in situ monitoring of the enzymatic reaction of the PCA or gallate biotransformation products was performed, for the first time, using a 5 mm NMR tube bioreactor [33,34]. For this, 1 mM of the substrate was added to 8 μM of PcaA in a 5 mm NMR tube and 1D and 2D NMR data were recorded. Figure 4a illustrates the progress of the enzymatic reaction 2 and 24 h after the addition of the PCA in the NMR tube. After 2 h, approximately 8% of PCA was converted to the 4-carboxy-2-hydroxymuconate-6-semialdehyde (CHMS) product (Figure 4ai), while 24 h later, the PCA was totally consumed by 4,5-dioxygenase (Figure 4aii). The 1D ^1^H NMR spectrum of CHMS exhibited a characteristic deshielded doublet resonance at 9.17 ppm (Figure 4b) which was attributed to the aldehyde proton H6e. Further, a doublet at 8.45 ppm was assigned to H5e and a singlet resonance at 8.84 ppm was attributed to H3e. It should be emphasized that the ^1^H NMR resonances of PcaA are invisible due to extensive line broadening and the very low concentration (8 μM) relative to that of PCA (1 mM). The control assay is the ^1^H NMR spectrum of the biotransformation of PCA by PcaA inside the NMR tube at 0 h (no observable signals of the biotransformation product, Figure S3), followed by the ^1^H NMR spectrum after 2 h of reaction (very weak signals of the reaction product, Figure 4a) and 24 h (Figure 4b)

Following the enzymatic reaction in the NMR tube also allowed to record, as is illustrated in Figure 4b, the keto-enol tautomerization, since two reaction products were unveiled. The keto form, which is the minor CHMS product, demonstrated a less deshielded resonance at 8.81 ppm due to H6k proton. Proton H5k showed a doublet at 7.98 ppm and the two H3k protons resonances appeared at 7.91 and 8.01 ppm. The excellent resolution and signal to noise ratio of the recorded 1D ^1^H spectra allowed the application of various 2D NMR experiments. The ^1^H-^1^H COSY NMR spectrum demonstrated a characteristic cross-peak between H5 and H6 protons for both the keto and enol forms. In the ^1^H-^1^H TOCSY NMR spectrum an intense cross-peak between H5 and H6 protons appeared for both the keto and enol form and two less intense cross-peaks between H3 and H5 protons and H3 and H6 protons for both tautomeric forms (Figure S4).

The 2D ^1^H-^13^C HSQC and HMBC NMR spectra allowed the complete assignment of the carbon chemical shifts for both the keto and enol forms. ^13^C chemical shifts at 144.55, 130.05, and 129.39 were attributed to C6, C5, and C3 of the enol form, respectively. The C6, C5, and C3 carbons of the keto form were assigned at 145.29, 124.37, and 126.23 ppm, respectively. Informative ^1^H-^13^C HMBC NMR cross-peaks were observed between H6e and C5 and C3, H3e and C5, and between H5e and C3 and C6 of the enol form. No cross-peaks were recorded in the ^1^H-^13^C HMBC spectrum for the keto form due to its low abundance in the enzymatic reaction.

**Figure 5:**
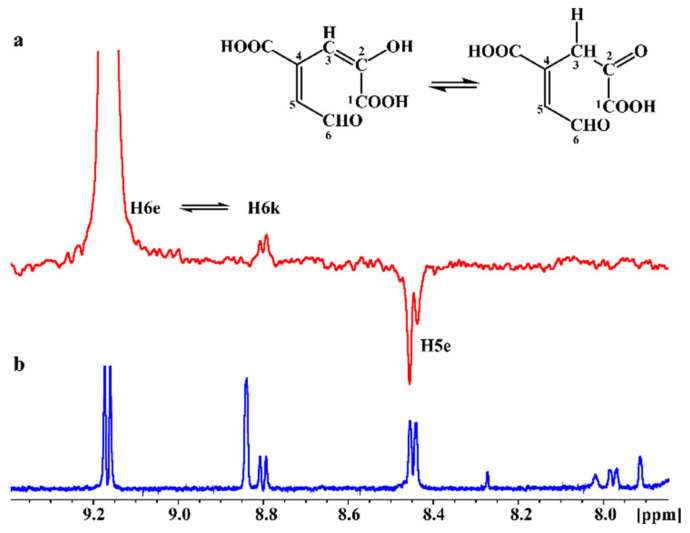
(**a**) STD NMR spectrum of the enzymatic biotransformation of PCA by 4,5-dioxygenase to the reaction product, CHMS, inside the NMR tube. Irradiation was applied selectively on the H6e proton resonance of the enol form. (**b**) ^1^H NMR spectrum of the same sample.

STD NMR methodology has previously been applied to simultaneously screen for interactions of the biotransformation products with protein targets in the NMR tube [33,34]. This NMR technique is based on the intermolecular transfer of magnetization from the irradiated protein receptor to the ligand resulting in the determination of interacting protons that are in proximity to the protein (≤5 Å) [35]. Herein, we applied the reverse effect and successfully irradiated the H6e proton of the enol form. Interestingly, magnetization was transferred not only to H5e proton of the same molecule via spin diffusion, through the nuclear Overhauser effect, which resulted in a negative peak, but also to the H6k proton of the keto tautomer which appeared as a positive peak, possibly due to the rapid interexchange of the enol-keto forms on the tautomerism (Figure 5). To the best of our knowledge, this is the first demonstration of such an effect with STD NMR methodology.

Finally, the equilibrium of the two tautomers was investigated by the use of variable temperature gradient ^1^H NMR spectroscopy in the buffer solution at pH 8. It should be noted that at the pH value used, the carboxylic groups are deprotonated. The keto/enol equilibrium of the product was described by the equilibrium constant K_eq_ as described in the Supplemental Material [36,37]. The two well-resolved resonances of H3e and H3k were recorded as the enol and keto equilibrium markers, respectively, over a sufficient range of temperatures (290–315 K), which allowed the accurate determination of lnK_eq_ (Figure S5) and, therefore, of ΔH° and ΔS°. The thermodynamic parameters were calculated ΔH° = 25.58 kJ/mol and ΔS° = 0.10 kJ/mol K. At 298 K, −TΔS° = −28.69 kJ/mol and the free energy difference ΔG° at 298 K was found to be −3.11 kJ/mol.

**Figure 6:**
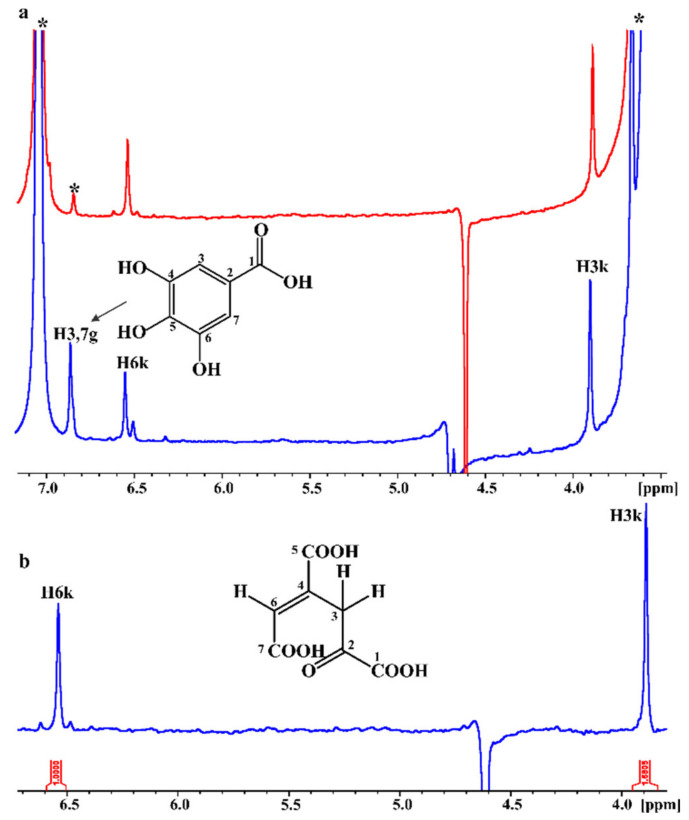
In situ monitoring of the biotransformation of gallate by PcaA, inside the NMR tube. (**a**) ^1^H NMR spectra 24 h (blue spectrum) and 48 h (red spectrum) after the addition of the substrate to PCA 4,5-dioxygenase. Proton NMR resonances of the substrate are shown in the blue spectrum. The asterisks (*) denote peaks of the Tris-buffer (3.5 ppm), the imidazole (7 ppm), and the carbon satellite of imidazole (6.8 ppm). (**b**) Expanded region of the ^1^H NMR spectrum of the OMA keto form enzymatic reaction products (the integrals of the three protons are presented) (number of scans = 64, acquisition time = 4 s, relaxation delay = 1.5 s, total experimental time = 6 min 22 s).

The progress of the biotransformation of gallate as the substrate after 24 and 48 h within the NMR tube is illustrated in Figure 6a. After 24 h, approximately, 59% of gallate was biotransformed, through ring-cleavage reaction to the 4-oxalomesaconic acid product, while 48 h later, all the substrate was consumed. Application of 1D (Figure 6b) and 2D NMR spectroscopy allowed the characterization of the keto form of the product in the NMR reaction tube. The peak at 6.54 ppm can be assigned to the olefinic H6k proton and the deshielded resonance at 3.89 ppm to the two H3k protons. Similarly, 2D ^1^H-^13^C HSQC and HMBC NMR spectra allowed the complete assignment of the chemical shifts of the carbons of the product. The resonances at 39.73, 132.7, 137.2, 174.7, and 201.9 ppm can be assigned to carbons C3, C6, C4, C5, and C2, respectively. Informative ^1^H-^13^C HMBC NMR cross-peaks were observed among H3k protons and C6, C4, C5, and C2 in the spectrum. For H6 proton, only cross-peaks with C3 and C5 were observed in the ^1^H-^13^C HMBC spectrum due to the low concentration of the product.

## 3. Discussion

We have formerly demonstrated that *P. phenanthrenivorans* Sphe3, isolated from a creosote-oil contaminated soil [24], degrades phenanthrene via *o*-phthalate, leading to the formation of PCA, which in turn could be metabolized via both 3,4- and 4,5-ring cleavage pathways [29]. In silico analysis of the Sphe3 genome has led to the annotation of all the putative genes involved in the 4,5-cleavage pathway and RT-PCR analysis has revealed that the genes constitute a single transcriptional unit; results that are consistent with those reported for *C. testosteroni* [15], *P. straminea* [17], and *A. keyseri* [30]. The gene organization of the PCA 4,5-cleavage pathway in Sphe3, as results from the comparison with other 4,5-gene clusters (Figure 2a), has been characterized as *Comamonas* type—consisting of a single transcriptional unit—in contrast to the *Sphingobium* type of organization, which appears to consist of several transcriptional units [38].

To date, the majority of the studied PCA 4,5-dioxygenases consist of a small *α* and a large *β* subunit in a heterotetrameric structure *α*_2_*β*_2_ as exemplified by LigAB of *C. testosteroni* enzyme crystal structure [39]; however, PmdAB from *C. testosteroni* T-2 has been reported to be a heteromultimer (*αβ*)_4_ [15], whereas a homodimer enzyme consisting of a single subunit has been reported for *Rhizobium leguminosarum* biovar *viceae* USDA 23780 [11].

**Figure 7:**
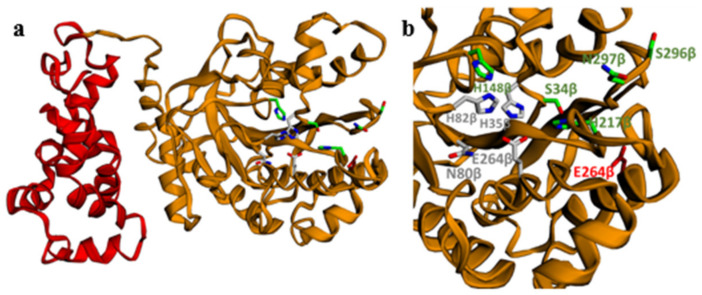
(**a**) The 3D structure of PcaA using homology modeling. Subunits *α* and *β* are colored in red and orange, respectively. Conserved residues H35β, N80β, H82β, and E264β are colored in gray and correspond to the iron-binding residues. The conserved residues S34β, H148β, H217β, S296β, and N297β that can interact specifically with the aromatic ring of PCA are colored in green. The highly conserved aspartate residue D322β is colored in red. (**b**) A closer view of subunit *β* with details on the conserved residues.

In contrast to the typical organization as a heterodimer, a fusion protein is encoded in the Sphe3 genome bearing the regions corresponding to the *α* and *β* subunits of PCA 4,5-dioxygenases and the predicted protein product PcaA demonstrated key conserved features of the enzyme as revealed by multiple sequence alignment (Figure S1). The structure of PcaA (Figure 7) was generated using homology modeling in Modeller [40]. Similarly to LigAB (pdbid: 1B4U) from *S. paucimobilis* SYK-6 [39], H35β, N80β, H82β, and E264β correspond to the iron-binding residues, while residues S34β, H148β, H217β, S296β, and N297β interact specifically with the aromatic ring of PCA (the Greek character after the residue number corresponds to the subunit of PcaA that each residue is located). The coordination geometry of the non-heme Fe (II) in PcaA should follow the coordination geometry of the non-heme Fe (II) in DesB (pdbid: 3WRB) [41] and LigAB (pdbid: 1B4U) (Figures S6–S8). The four conserved histidine residues are found in four known motifs: SHXPA (H35β in PcaA) and NDHA (H80β in PcaA) in iron coordination, and DHG (H148β in PcaA) and GXSH (H217β in PcaA), which are characteristic of the novel PCAD-Memo superfamily [14,15,42,43]. An absolutely conserved aspartate residue is also present (D232β in PcaA) providing a stabilizing backbone contact that is crucial for delimiting the one side of the active site [14]. Contiguous DNA segments joined to form a single gene encoding for the large and small subunit of PCA 4,5-dioxygenases have been reported for *Arthrobacter keyseri* [30], *Verminephrobacter eiseniae* EF01-2, and *Burkholderia phymatum* STM815 [44].

Transcriptional analysis showed that the *pca*A gene encoding the 4,5-PCA dioxygenase was induced during growth on PCA and phenanthrene. The higher *pca*A mRNA transcript level upon PCA growth in comparison with the phenanthrene-grown cells could be attributed to a different regulatory mechanism, triggered by the presence of different carbon sources or even to the higher growth rate of Sphe3 in PCA compared to phenanthrene that could affect the *pca*A transcription [45,46]. Similar results were obtained by proteomic analysis of Sphe3 cells grown on phenanthrene or on phthalate, an intermediate metabolite of phenanthrene metabolism pathway [29].

The divergently directed LysR-type transcriptional regulator (LTTR) (*pca*R), located upstream of the *pca* gene cluster in Sphe3, was induced in the presence of aromatics, with a 6-fold higher induction for PCA. The minimal expression of *pca*R gene during growth in the presence of glucose indicates a minimal basal constitutive expression, but in response to PCA or a metabolite thereof that act as inducers, PcaR can act as the transcriptional activator of the PCA *meta*-cleavage pathway in Sphe3, as has been reported for LTTRs in aromatic degradation pathways [47,48,49]. PcaR shows no similarity to any characterized LTTR but shares a 97% amino acid sequence identity with *pcm*R found upstream the *pmc* gene cluster in *A. keyseri* and whose role in regulation has not been studied [30]. Studies on the regulatory mechanisms of the PCA 4,5-cleavage pathway are scarce [5,8,38] and given the fact that specific regulators among each bacterial group of Actinobacteria, α-, β- and γ-Proteobacteria diversely control transcription of PCA gene clusters [8], the study of the regulation of the PCA *meta*-cleavage pathway in Sphe3 in concert with the LysR-type protein and the possible influence of other regulatory elements remains to be clarified in the future.

To the best of our knowledge, this is the first report on biochemical and kinetic characterization of a PCA 4,5-dioxygenase in the genus *Arthrobacter*. PCA 4,5-dioxygenase from *P. phenanthrenivorans* Sphe3 (PcaA) was overexpressed, purified, and characterized for its biochemical and kinetic properties.

The highest activity for the recombinant PCA 4,5-dioxygenase was recorded after 30 min of incubation in the presence of a 0.2 mM FeSO_4_/L-ascorbic acid solution at 0 °C. Like other type II extradiol dioxygenases, PCA 4,5-dioxygenase seems to depend on the presence of Fe(II) as a co-factor that is involved in the catalysis [13]. The apparent *K_m_* value of PcaA for PCA is lower than or similar to values reported for 4,5-dioxygenases from other organisms, even under different experimental conditions in some cases (80 μM, *Pseudomonas* sp. [32]; 55.5 μM, *C. testosteroni* T-2 [15]; 46 μM, *C. testosteroni* NCIMB 8893 [50]; 20 μM, *R. leguminosarum* [11]; 51 ± 4 μM, *S. paucimobilis* SYK-6 [12]; 5.26 μM, *R. ruber* P25 [31]), revealing a high affinity with PCA as a substrate. In contrast to the majority of extradiol ring-cleavage dioxygenases containing iron Fe(II) whose activity is decreased by the addition of chelators [12,13,51], the activity of PcaA is not significantly reduced in the presence of metal chelating agents (EDTA, bipy, *o*-phenanthroline). This indicates either that Fe(II) is tightly bound to the iron center of PcaA or that the latter is not easily accessible [12].

Non-heme iron extradiol dioxygenases have been reported to be catalytically active most commonly with Fe(II) but also with a variety of metal ions of the same oxidation state (e.g., Mn(II), Co(II), Cu(II), Zn(II) or Ni(II)) [51,52,53]. Of the divalent metal ions tested in the present study, only Cu(II) and Mn(II) were inhibitory and the same inhibitory effects of Cu(II) and Mn(II) have been also reported for other PCA 4,5-dioxygenases [12,50], allowing the assumption that they may out-compete Fe(II) for the occupation of the metal ion binding site. The optimum pH for PcaA catalysis is 9.5, which is consistent for PCA 4,5-dioxygenase of *Pseudomonas* sp. K82 and *R. leguminosarum* [11,54], whereas most of the bacterial PCA 4,5-dioxygenases have been reported to show the highest activity in a pH range of 7–8 [12,17,32].

The narrow range of substrates catabolized by 4,5-PCA dioxygenase in Sphe3 denotes that the enzyme is highly specific to PCA. The lack of enzyme activity in the presence of homoprotocatechuate indicates that the carboxylate group is essential for the aromatic ring cleavage and cannot be replaced by a carboxymethyl group [55]. Additionally, the vicinal 3,4-diol has been considered crucial for the enzyme activity as benzoate, 4-hydroxybezoate, and 3-hydroxybenzoate showed no activity results that corroborate those reported for PmdAB from *C. testosteroni* T-2 [15] and LigAB from *S. paucimobilis* SYK-6 [12].

NMR analysis clearly illustrated the presence of two PCA biotransformation products due to keto-enol tautomerization, which has been previously reported to exist spectrophotometrically [56,57], whereas NMR resonances corresponding to the biotransformed product have not formerly been observed in the ^1^H NMR spectrum [12]. An unequivocal confirmation of the tautomerization process was further confirmed with STD NMR. The formation of the keto tautomer was enthalpically favored, presumably due to hydrogen bonding to the carbonyl group; however, it was strongly entropically disfavored, thus resulting in a shift of the equilibrium towards the formation of the dominant enol form. It is well known that the keto-enol equilibrium in solution is very sensitive and related to polar/polarizability and hydrogen-bonding capability of solvents [58]. The enol tautomer was entropically favored and stabilized due to weaker intermolecular hydrogen bonding formation with the aqueous solvent and, thus, the least immobilization of the solvation molecules of H_2_O. Since a buffer solution was used, it can be concluded that there is no change in the ionization state of the keto and enol tautomers due to temperature variation. Additionally, the OMA product resulting from the biotransformation of gallate has previously been characterized only by ^1^H NMR spectroscopy; however, the reaction was not monitored inside the NMR tube [59].

^1^H [60,61], ^13^C [62], ^19^F [63], and ^31^P [64,65] NMR has emerged as a powerful and reliable tool for the investigation of products of enzymatic reactions and for gaining insight into the kinetics of the reactions. More specifically, in situ NMR diffusion experiments were explored for their ability to profile reaction products with significantly different molecular weight over the course of enzymatic depolymerization of biological macromolecules [66]. ^19^F and ^31^P NMR require the presence of the appropriate isotope in the ligands. ^13^C NMR requires selectively enrichment in ^13^C ligands and/or the use of hyperpolarization methods which require specialized NMR instrumentation [67]. The advantage of the present in situ monitoring approach of an enzymatic reaction by free enzyme in the NMR tube is that the complete characterization can be achieved by the combined use of 1D, 2D, ^1^H-^1^H, and ^1^H-^13^C HSQC and HMBC NMR spectroscopy. Furthermore, various restriction factors, including enzyme loss of activity and stability, substrate concentration limitations, temperature, and buffer limitations, made this study more challenging. However, we successfully performed the reaction in optimum conditions, monitored the reaction over time, and elucidated the structural components of the products. A basic advantage of the present method is that the enzyme is free in the solution and, therefore, it can adopt its natural conformation, which is an essential factor for a natural enzymatic biotransformation as a dynamic process. Furthermore, this approach minimizes the need for laborious sample preparation methods and purification steps, ultimately leading to more accurate real-time kinetic determination of enzymatic reactions.

## 4. Materials and Methods

### 4.1. Bacterial Strains and Growth Conditions

*P. phenanthrenivorans* Sphe3^T^ (formerly *Arthrobacter phenanthrenivorans* Sphe3 = DSM 18606^T^ = LMG 23796^T^) was isolated from a creosote-polluted area in Epirus, Greece [23]. Sphe3 was grown in M9 minimal medium (MM M9), as described previously [68], in the presence of phenanthrene (15 mM), PCA (5 mM) or glucose (22 mM) as the sole carbon and energy sources, on a rotary shaker agitated at 180 rpm, at 30 °C. *Escherichia coli* DH5a and BL21(DE3) were cultured in lysogeny broth (LB) medium in the presence of Kanamycin (Km) when necessary, for selection.

### 4.2. Preparation of Sphe3 Cell Extracts

Crude cell-free extracts were prepared as described previously [68] with a slight modification in the washing buffer (10 mM Tris-HCl, pH 8.0 containing 10 mM DTT). Protein concentrations were determined spectrophotometrically by the method of Bradford, using the Bio-Rad reagent (Bio-Rad Laboratories, Hercules, CA, USA) and bovine serum albumin (BSA) (Amresco Inc., Solon, OH, USA) as a standard [69].

### 4.3. Enzyme Assays

The activity of PCA 4,5-dioxygenase under standard conditions was estimated spectrophotometrically as described by Ono et al. [32] with a slight modification in the reaction buffer (100 mM Glycine-NaOH, pH 9.5). The reaction was initiated with the addition of the crude cell-free extract (8 mg/ml) or the recombinant purified PcaA solution (0.15 mg/ml). One enzyme unit is defined as the amount of enzyme that produces 1 μmol of 2-hydroxy-4-carboxymuconate-6-semialdehyde per minute at 20 °C. The kinetic parameters *K_m_* and *V_max_* values were determined in a broad concentration range of PCA substrate varying from 0.015 to 15 mM, with Origin Microcal software, Version 6.0, ORIGIN [70], by nonlinear fitting of the Michaelis–Menten equation. All kinetic values are the means of three separate measurements. The effect of pH in the activity of recombinant PcaA was investigated in the pH range of 4 to 12 at room temperature. The buffer solutions (100 mM) used to span this range were succinate-NaOH (pH 4.0 to 6.0), citrate-NaOH (pH 6.5), phosphate (pH 7.0), Tris (pH 7.5 to 9), and glycine (pH 9.5 to 12). The optimal temperature for the PCA 4,5-dioxygenase was determined by conducting the above assays in a temperature range of 5 to 50 °C in the optimal pH.

The influence of different chelating agents on the PcaA activity was tested by incubating samples of the purified enzyme (1 μM) with concentrations of EDTA, 2,2′-dipyridyl (2,2′-bipyridine) (bipy), or *o*-phenanthroline up to 10 μM at 0 °C for 30 min in 100 mM glycine-NaOH buffer pH 9.5. The enzyme reaction was initiated by adding 0.3 mM PCA.

The influence of different divalent metals on PcaA activity was estimated spectrophotometrically as above. The enzyme was incubated with concentrations of divalent metal salt solutions of FeSO_4_·7H_2_O/L-ascorbic acid, CuSO_4_·5H_2_O, MgSO_4_·7H_2_O, MnSO_4_·H_2_O, and NiCl_2_·6H_2_O, ranging from 0.1–1 mM and for time periods of 0, 15, 20, and 30 min at 0 °C.

All enzyme assays were performed at 20 °C using a Shimadzu UV-1201 spectrophotometer (Triad Scientific, Inc., Manasquan, NJ, USA).

### 4.4. Substrate Specificity Assessment

A variety of potential substrates were screened by measuring O_2_ consumption using an O_2_-sensitive Clark type electrode with computer integration via an Oxygraph electrode control unit (HI 9143 Microprocessor Auto Cal Dissolved Oxygen Meter, HANNA instruments, Smithfield, RI, USA). The reaction mixture contained 0.3 mM of the tested substrate solution, 0.15 mg/ml of purified enzyme, and 100 mM glycine-NaOH buffer pH 9.5 to a final volume of 2 mL. Recombinant PcaA was added in order to initiate the reaction as previously described. Stock solutions of the organic substrates were prepared as 30 mM solutions in H_2_O (PCA, gallate, 4-hydroxybezoate, 3-hydroxybenzoate, gentisate, phthalate, catechol, 3,4-dihydroxyphenylacetate), 1:9 DMSO/H_2_O (benzoate, vanillin), or as 10 mM solutions in 1:4 DMSO/H_2_O (caffeic acid, *p*-coumaric acid).

### 4.5. Cloning and Heterologous Expression of pcaA Gene

The *orf* identified from in silico analysis of the Sphe3 genome that encodes the putative *α* and *β* subunits of PCA 4,5-dioxygenase was found on an indigenous plasmid (pASPHE302) and annotated as *pca*A. Sphe3 genomic DNA was isolated from phenanthrene grown cells using cetyltrimethylammonium bromide (CTAB) [71]. Primers Pca45diox_for_/Pca45diox_rev_ (Table S1) carrying *Nde*I-*Xho*I restriction sites and gDNA as template were used in a PCR reaction with conditions as described previously [68]. The 1302 bp amplification product was cloned in pCR-blunt vector (Invitrogen, Thermo Fischer Scientific, Watham, MA, USA) for nucleotide sequence verification and subsequently subcloned with *Nde*I/*Xho*I restriction in the overexpressing vector pET29c_(+)_ (Novagene Co., Ltd., Beijing, China).

Amplification reactions were carried out in a PTC-100 version 7.0 thermocycler (MJ Research Inc., Waltham, MA, USA) using the Phusion High-Fidelity DNA polymerase (Finnzymes, Espoo, Finland) and 1.5 mM MgCl_2_ (final concentration). NucleoSpin Extract 2 in 1 (Macherey-Nagel GmbH & Co. KG, Dueren, Germany) was used to purify the produced amplicons.

For *E. coli* strain DH5a as a recipient, host transformation was carried out according to Hanahan [72] and for BL21 (DE3) according to Chung and Miller [73]. Cloned fragments were sequenced by VBC Genomics Bioscience Research GmbH (Wien, Germany). Sequence alignments were performed using the program BLAST [74] at the NCBI website. Restriction enzyme digestions, ligation, and agarose gel electrophoresis were carried out using standard methodology [75]. Amino acid sequences exhibiting similarity were retrieved from protein databases and aligned using CLUSTALW (accessed on 5/4/2021) [76]. Phylogenetic tree and molecular evolutionary analyses were conducted using MEGA version 4 with the neighbor joining method [77].

### 4.6. Purification of Recombinant PCA 4,5-Dioxygenase

An *E. coli* BL21(DE3)-pET29c_(+)_ host–vector overexpression system was used to overproduce the PcaA polypeptide as described elsewhere [68].

The supernatant was applied to a 20 mL HisPrep FF 16/10 column (GE Healthcare, Chicago, IL, USA) using and AKTA FPLC system (GE Healthcare, Chicago, IL, USA). Column bound protein was washed with wash buffer (100 mM Tris-HCl, 500 mM NaCl, pH 8.0 containing 25 mM imidazole) and then eluted with elution buffer (100 mM Tris-HCl, 500 mM NaCl, pH 8.0 containing 350 mM imidazole). The elution buffer was exchanged with NMR buffer (50 mM Tris-HCl, 50 mM NaCl, 250 μΜ DTT pH 8) and 10% DMSO-*d_6_* was added for better enzyme stability.

### 4.7. In situ Biotransfomation Monitoring in the NMR Tube

First, 1 mM of PCA was added to 8 μM of enzyme in 0.6 mL of NMR buffer 90% H_2_O (50 mM Tris-HCl, 50 mM NaCl, 0.25 mM DTT, pH 8) with 10% DMSO-*d_6_*, then the sample was transferred to a 5 mm NMR tube. Similarly, 0.5 mM of gallate was added to 8 μM of enzyme in NMR buffer 90% H_2_O 10% DMSO-*d_6_* and transferred to a 5 mm NMR tube. NMR experiments were performed directly in the NMR tube and the enzymatic reaction was monitored over time. NMR experiments were performed on a Bruker AV500 spectrometer (Bruker Biospin, Rheinstetten, Germany) at 298 K using the Topsin 3.2 suite. Water suppression was achieved by the use of an excitation sculpting pulse sequence (zgesgp). The assignment and the description of the physicochemical properties of the reaction products was achieved using 1D ^1^H and 2D NMR spectroscopy, ^1^H-^1^H COSY, ^1^H-^1^H TOCSY, ^1^H-^13^C HSQC, and ^1^H-^13^C HMBC experiments. The 2D TOCSY NMR experiment was acquired using a mixing time of 60 ms. Saturation transfer difference (STD) NMR experiment was performed with the selective saturation of a given ^1^H frequency by a train of 40 Gaussian pulses with the duration of 50 ms each separated by a delay of 1 ms, with total saturation time of 2 s. The on-resonance irradiation was performed at a chemical shift of 9.17 ppm. Off-resonance irradiation was applied at 40 ppm, outside the spectra region of the ^1^H NMR resonances. STD spectrum was obtained by the subtraction of the on-resonance from the off-resonance spectrum.

### 4.8. Transcriptional Analysis with RT-PCR and Quantitative Real-Time PCR (RT-qPCR)

Total RNA isolation was performed as described elsewhere [32]. For RT-PCR analysis the Robust^TM^ II RT-PCR Kit (Finnzymes Oy, Thermo Fischer Scientific, Espoo, Finland) was used according to the manufacturer’s instructions and primers (Table S1) designed to amplify the six gene junctions (Figure S1A). RT-qPCR was performed as described previously [68] with a modification in cDNA synthesis, where the PrimeScript^TM^ RT reagent kit with gDNA eraser (Perfect Real Time, Takara Bio Inc., Kusatsu, Shiga, Japan) was used according to the manufacturer’s instructions.

The efficiency (E) of one cycle RT-qPCR in the exponential phase was found to be 1.89–2.2 (E = 10^(−1/slope)^ [78,79] with correlation factors 0.9894 < *R^2^ <* 0.9918). The housekeeping gene was used as the reference gene and gene expression levels in glucose were used as calibrator *gyr*β. The results were analyzed by the relative quantification method [80].

### 4.9. 3D Model Construction of PcaA

The 3D model of PcaA was generated by homology modeling, using standard routines in the MODELLER 10.0 software [40]. Three known structures with pdbids, 1B4U, 3WRB, and 3MQU, were used as templates. The pairwise alignment of the PcaA sequence with the sequences of known structures used for the modeling can be found in Supplementary Material (embedded alignment.pdf accessed on 8 August 2021). The generated model was visualized with EzMol [81]. The figures of the non-heme Fe (II) site of 3WRB and 1B4U were generated with Mol*Viewer 2.2.1 (https://molstar.org/viewer/, accessed on 8 August 2021) [82].

## Data Availability

All data generated or analyzed during this study are included in this published article (and its Supplementary Information File).

## Supplementary Materials

The following are available online at https://www.mdpi.com/article/10.3390/ijms22179647/s1.
