# Supplementary material for: Characterization of Protocatechuate 4,5-Dioxygenase from Pseudarthrobacter phenanthrenivorans Sphe3 and In Situ Reaction Monitoring in the NMR Tube
Source: Int J Mol Sci. 2021 Sep 6;22(17):9647. doi: 10.3390/ijms22179647 (PMC8431788; doi:10.3390/ijms22179647)
Supplement: Supplementary file 1 [file ijms-22-09647-s001.zip › ijms-1313856-embedded.pdf]

>PcaA
MTLDKPYNDVPGTTIFDADQARKGYNLNQFCMSLMKPENRERYLADRGAYLDEWPLNPVQRQ
AVLDIDLNTCIAEGGNIYFLAKIGATHGLSFQQMAGSM
TGMSEAAYRDMMIGGGRRPEGNRLKDLDGWTPPEPGEKAETVRQDAPAQYTSALFTSHVPAIG
AAMDLGKTEEPYWKKVFSGYEWTREWAKE--NLPDVV
ILVYNDHATAFDSSIIPTFVLGTGAEYPVADEGYGPRPVPDVKGYPELAAHIAQSVIQDDFDLTLV
NEMVVDHGLTVPLSLVYGDVEEWPVRVIPLAVNV
VQYPVPSGRRCYELGRALRRALDKWDGEPLNVQIWGTGGMSHQLQGPRAGLINEEWDNAFLD
HLIADPVGLTEWQHMEYVDEAGSEGIELVDWLIARGAM
DDQFGGESPEVNHRFYHVPASNTAVGHLVLTNQTD
>1B4U

ARVTTGITSSHIPALGAAIQTGTSDNDYWGPVFKGYQPIRDWIKQPGNMPDVV
ILVYNDHASAFDMNIIPTFAIGCAETFKPADEGWGPRPVPDVKGHPDLAWHIAQSLILDEFDMTI
MNQMDVDHGCTVPLSMIFGEPEEWPCKVIPFPVNV
VTYPPPSGKRCFALGDSIRAAVESFP-
EDLNVHVWGTGGMSHQLQGPRAGLINKEFDLNFIDKLISDPEELSKMPHIQYLRESGSEGVELVM
WLIMRGAL
P-----EKVRDLYTFYHIPASNTALGAMILQPE--

>pcaA
MTLDKPYNDVPGTTIFDADQARKGYNLNQFCMSLMKPENRERYLADRGAYLDEWPLNPVQRQ
AVLDIDLNTCIAEGGNIYFLAKIGATHGLSFQQMAGSM
TGMSEAAYRDMMIGGGRRPEGNRLKDLDGWTPPEPGEKAETVRQDAPAQYTSALFTSHVPAIG
AAMDLGKTEEPYWKKVFSGYEWTREWAKENLPDVVIL
VYNDHATAFDSSIIPTFVLGTGAEYPVADEGYGPRPVPDVKGYPELAAHIAQSVIQDDFDLTLVN
EMVVDHGLTVPLSLVYGDVEEWPVRVIPLAVNVVQ
YPVPSGRRCYELGRALRRALDKWDGEPLNVQIWGTGGMSHQLQGPRAGLINEEWDNAFLDHLI
ADPVGLTEWQHMEYVDEAGSEGIELVDWLIARGAMDD
QFGGESPEVNHRFYHVPASNTAVGHLVLTNQTD
>1B4U

EFDDIPGTRVFTAQRARKGYNLNQFAMSLMKAENRERFKADESAYLDEWNLTPAAKAAVLARD
YNAMIDEGGNVYFLSKLFSTDGKSFQFAAGSM
TGMTQEEYAQMMIDGGRSPAGVR---

>PcaA

MTLDKPYNDVPGTTIFDADQARKGYNLNQFCMSLMKPENRERYLADRGAYLDEWPLNPVQRQ
AVLDIDLNTCIAEGGNIYFLAKIGATHGLSFQQMAGSM
TGMSEAAYRDMMIGGGRRPEGNRLKDLDGWTPPEPGEKAETVRQDAPAQYTSALFTSHVPAIG
AAMDLGKTEEPYWKKVFSGYEWTREWAKENLPDVVIL
VYNDHATAFDSSIIPTFVLGTGAEYPVADEGYGPRPVPDVKGYPELAAHIAQSVIQDDFDLTLVN
EMVVDHGLTVPLSLVYGDVEEWPVRVIPLAVNVVQ
YPVPSGRRCYELGRALRRALDKWDGEPLNVQIWGTGGMSHQLQGPRAGLINEEWDNAFLDHLI
ADPVGLTEWQHMEYVDEAGSEGIELVDWLIARGAMDD
QFGGESPEVNHRFYHVPASNTAVGHLVLTNQTD
>3WRB

AKIIGGFAVSHTPTIAFAHDANKYDDPVWAPIFQGFEPVKQWLAEQKPDVTFY
VYNDHMTSFF-
EHYSHFALGVGEEYSPADEGGGQRDLPPIKGDPELAKHIAECLVADEFDLAYWQGMGLDFGAFS
PLSVLLPHEHGWPCRIVPLQCGVLQ
HPIPKARRFWNFGRSLRRAIQSYP-
RDIKVAIAGTGGLSHQVHGERAGFNNTEWDMEFMERLANDPESLLGATVTDLAKKGGWEGAE
VVMWLLMRGALS-
----PEVKTLHQSYFLP-SMTAIATMLFEDQGD

>PcaA
AEGGNIYFLAKIGATHGLSFQQMAGSM--TGMSEAAYRDMMIGGGRRP---EGNRLKDLD
GWTPPEPGEKAETVRQ
>3MQU
ALTDALYFIXGSFVEAGLEPGPLFEIVQQANXAK------LGPDGQPIFRESDQKVXKPD
GWLPPEPQLEAEVVRQ
