# Supplementary material for: Characterization of Protocatechuate 4,5-Dioxygenase from Pseudarthrobacter phenanthrenivorans Sphe3 and In Situ Reaction Monitoring in the NMR Tube
Source: Int J Mol Sci. 2021 Sep 6;22(17):9647. doi: 10.3390/ijms22179647 (PMC8431788; doi:10.3390/ijms22179647)
Supplement: Supplementary file 1 [file ijms-22-09647-s001.zip › ijms-1313856-supplementary.pdf]

SUPPLEMENTARY MATERIAL

Characterization of Protocatechuate 4,5-Dioxygenase from *Pseudarthrobacter phenanthrenivorans* Sphe3 and *in situ* reaction monitoring in the NMR tube.

Epameinondas Tsagogiannis[1], Elpiniki Vandera[1#], Alexandra Primikyri[2#], Stamatia Asimakoula[1], Andreas Tzakos[3], Ioannis Gerothanassis[2] and Anna-Irini Koukkou[1]*

1. Laboratory of Biochemistry, Sector of Organic Chemistry and Biochemistry, Department of Chemistry, University of Ioannina, 45110 Ioannina, Greece

2. Laboratory of Organic Chemistry, Sector of Organic Chemistry and Biochemistry, Department of Chemistry, University of Ioannina, 45110 Ioannina, Greece

[#]Equal contribution

*Address correspondence to:

Anna-Irini Koukkou, e-mail: akukku@uoi.gr; Tel: +30- 2651008371

A

```
LigA            MTEKKERIDVHAYLAEFDDIPGTRVFTAQRARKGYNLNQFAMSLMKAENRERFKADESAY 60
PcaA            ----------MTLDKPYNDVPGTTIFDADQARKGYNLNQFCMSLMKPENRERYLADRGAY 50
PcmA            ----------MTLDKPYKDVPGTTIFDADQARKGYNLNQFCMSLMKPENRERYLADRGAY 50
PmdA-T2         ----------MALEKPYLDVPGTIIFDAEQSRKGYWLNQFCMSLMKAENRERFRADERAY 50
ProOa           ----------MALEKPYLDVPGTIIFDAEQSRRGYWLNQFCMSLMKAENRDRFRANERAY 50
LigA-Rp         ----------MPLDKPYKDVPGTTIFDADMSRQGYHLNQFCMSLMKAENRARFKADERAY 50
                                : *:*** :* *: :*:** ****.***** *** *: *:. **

                             #            #
LigA            LDEWNLTPAAKAAVLARDYNAMIDEGGNVYFLSKLFSTDGKSFQFAAGSMTGMTQEEYAQ 120
PcaA            LDEWPLNPVQRQAVLDIDLNTCIAEGGNIYFLAKIGATHGLSFQQMAGSMTGMSEAAYRD 110
PcmA            LDEWPLNPVQRQAVLDIDLNTCIAEGGNIYFLAKIGATHGLSFQQMAGSMTGMSEAAYRD 110
PmdA-T2         LDEWAMTEEQKQAVLARDLNWCMRTGGNIYFLAKIGATDGKSFQQMAGSMTGMTEEEYRA 110
ProOa           LDEWAMTEEQKQAVLARDLNWCMRTGGNIYFLAKIGATDGKSFQQMAGSMTGMTEEEYRA 110
LigA-Rp         LDEWPMTEEQKQAVLDRDLNRCIALGGNIYFLAKIGATDGKSFQQMAGSMTGMTEEEYRN 110
                **** :.   : ***  * *  :  ***:***:*: :*.* ***  *******::  *
                                                          A

LigA            MMIDGGRSPAGVRSIKGGY--------------------   139
PcaA            MMIGGGRRPEGNR--------------------------   123
PcmA            MMIGGGRRPEGNR--------------------------   123
PmdA-T2         MMMGGGRSAEGNRYVGEDGDAQAHHQPQGSAGNQNKEGN   149
ProOa           MMMGGGRSAEGNRYVGEDGDAQAHHQPQGSAGNQNKEGN   149
LigA-Rp         MMVGGGRPIEGNRYVGEKK--------------------   129
                **:.***   * *
```

B

```
                                                  #
LigB            -----------------------MARVTTGITSSHIPALGAAIQTGTSDNDYWGPVFKGY 37
PcaA            LKDLDGWTPPEPGEKAETVRQDAPAQYTSALFTSHVPAIGAAMDLGKTEEPYWKKVFSGY 60
PcmA            LKDLDGWTPPEPCEKAETVRQDAPAQYTSALFTSHVPAIGAAMDLGKTEEPYWKKVFSGY 60
PmdB-T2         -----------------------MARITASVFTSHVPAIGAAMDMGKTQEAYWAPLFKGY 37
ProOb           -----------------------MARITASVFTSHVPAIGAAMDMGKTQEAYWAPLFKGY 37
LigB-Rp         -----------------------MARITASVYTSHVPAIGAAIDMQKTGEDYWKPVFKGY 37
                                        *: *:.: :**:**:***::  .: : **  :*.**
                                                 PFAA

                                     # #
LigB            QPIRDWIKQPGNMPDVVILVYNDHASAFDMNIIPTFAIGCAETFKPADEGWGPRPVPDVK 97
PcaA            EWTREWAKE--NLPDVVILVYNDHATAFDSSIIPTFVLGTGAEYPVADEGYGPRPVPDVK 118
PcmA            EWTREWAKE--NLPDVVILVYNDHATAFDSSIIPTFVLGTGAEYPVADEGYGPRPVPDVK 118
PmdB-T2         DFSRQWMKD--NKPDVIFLVYNDHATAFSLDCIPTFAIGTAAEFQPADEGWGPRPVPKVV 95
ProOb           DFSRQWMKD--NKPDVIFLVYNDHATAFSLDCIPTFAIGTAAEFQPADEGWGPRPVPKVV 95
LigB-Rp         EFSKQWLKD--QKPDVIFLVFNDHATAFSLDLIPTFAIGTAAEYQPADEGWGPRPVPKVI 95
                :  ::* *:  : ***::**:****:**. . ****.:* .  :  ****:******.*
                                     F F

LigB            GHPDLAWHIAQSLILDEFDMTIMNQMDVDHGCTVPLSMIFGEP----EEWPCKVIPFPVN 153
PcaA            GYPELAAHIAQSVIQDDFDLTLVNEMVVDHGLTVPLSLVYGDV----EEWPVRVIPLAVN 174
PcmA            GYPELAAHIAQSVIQDDFDLTLVNEMVVDHGLTVPLSLVYGDV----EEWPVRVIPLAVN 174
PmdB-T2         GHPDLASHIAQSVIQQDFDLTIVNKMDVDHGLTVPLSLMCGEQDPKTGSWPCPVIPFAVN 155
ProOb           GHPDLASHIAQSVIQQDFDLTIVNKMDVDHGLTVPLSLMCGEQDPKTGSWPCPVIPFAVN 155
LigB-Rp         GHPRLAAHIAQSVIQDDFDLTIVNKMDVDHGLTVPLSLMCGEP----QAWPCPVIPFAVN 151
                *:* ** *****:* ::**:*::*:* **** *****:: *:       **  ***: **
                                             P

                                                                         ◆
LigB            VVTYPPPSGKRCFALGDSIRAAVESFPE-DLNVHVWGTGGMSHQLQGPRAGLINKEFDLN 212
PcaA            VVQYPVPSGRRCYELGRALRRALDKWDGEPLNVQIWGTGGMSHQLQGPRAGLINEEWDNA 234
PcmA            VVQYPVPSGRRCYELGRALRRALDKWDGEPLKVQIWGTGGMSHQLQGPRAGLINEEWDNA 234
PmdB-T2         VVQYPVPTGQRCFNLGRAIRKAVESYDQ-DINVHIWGTGGMSHQLQGARAGLINKEWDNQ 214
ProOb           VVQYPVPTGQRCFNLGRAIRKAVESYDQ-DINVHIWGTGGMSHQLQGARAGLINKEWDNQ 214
LigB-Rp         VVQYPVPSGRRCYMLGQAIGRAIRSYDE-DLNVQIWGTGGMSHQLQGPRAGLINREWDNA 210
                ** ** *:*:**: ** ::  *: .:    ::*::************ ******.*:*
                                                          A A
                                                          P

                                             #
LigB            FIDKLISDPEELSKMPHIQYLRESGSEGVELVMWLIMRGALPEKV------RDLYTFYHI 266
PcaA            FLDHLIADPVGLTEWQHMEYVDEAGSEGIELVDWLIARGAMDDQFG-GESPEVNHRFYHV 293
PcmA            FLDHLIADPVGLTEWQHMEYVDEAGSEGIELVDWLIARGAMDDQFG-GESPEVNHRFYHV 293
PmdB-T2         FLDLLVENPHGLAQMPHIDYVREAGSEGIELVMWLIARGAMSDVDGPAPLPKVAHRFYHV 274
ProOb           FLDLLVENPHGLAQMPHIDYVREAGSEGIELVMWLIARGAMSDVDGPAPLPKVAHRFYHV 274
LigB-Rp         FLDQIINDPDGLSQKPHIDYVREAGSEGIELVMWLIARGAMSDVAG-GPKPKVAHRFYHV 269
                *:* :: :*  *::  *::*: *:****:*** *** ***: :        .  : ***:
                                             F

LigB            PASNTALGAMILQPEETAGTPLEPRKVMSGHSLAQA       302
PcaA            PASNTAVGHLVLTNQTD-------------------       310
PcmA            PASNTAVGHLVLTNPTD-------------------       310
PmdB-T2         PASNTAVGHLILENQ---------------------       289
ProOb           PASNTAVGHLILENQ---------------------       289
LigB-Rp         PASNTAVGHLILENA---------------------       284
                ******:* ::*
                  PP
```

Supplementary Fig. S1: Multiple sequence alignment. Alignment (Clustal W) of the *α* (**A**) and *β* (**B**) subunits of PCA 4,5-dioxygenase (PcaA) from *P. phenanthrenivorans* Sphe3 with other PCA 4,5-dioxygenases of >60% identity. Although PcaA is a monomer, herein was considered as two subunits in order to facilitate the sequence comparisons. The linker sequence is underlined at the N terminus of the *β* subunit. Preserved histidine residues in all class II dioxygenases are also depicted in boldface. *Grey highlighting* indicates signature sequences for iron ligands observed in crystals of LigAB [39] . *Letters under the sequence* indicate residues as follows: *A*, nonspecific hydrophobic interaction with PCA; *P*, specific interaction with hydroxyl or carboxyl moieties of PCA; *F*, iron-atom coordination; *asterisks* conserved residues; *colons* conservative replacements. *Crosshatches* in A represent amino acids involved in the catalysis while in B they represent iron ligands identified for LigB [39]. The *black diamond* in B represents an absolutely conserved aspartate which provides a backbone contact crucial for delimiting the one side of the active site [14]. Sequences aligned: LigAB, from *S. paucimobilis* SYK-6 (M34835 [20]), PcaA (ADX75303.1, this study), PcmA from *A. keyseri* (AAK16524.1[30]), PmdAB-T2 from *C. testosteroni* (AF459635 [15]), ProOab from *P. straminea* (AB127969[17] and LigAB-Rp from *R. palustris* [WP_011160233.1, WP_011160234.1].

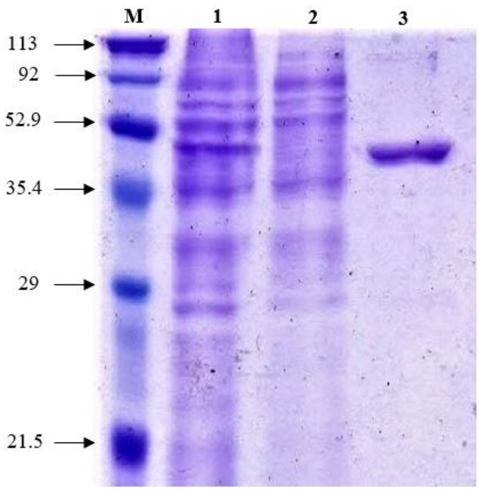

Supplementary Fig. S2: SDS-PAGE protein analysis. Purification of overexpressed PcaA from *P. phenanthrenivorans* Sphe3. Lane M, protein molecular mass marker in kDa; lane 1, cell-free crude extract from *E. coli* BL21/ pET29c::*pca*A; lane 2, Ni^{+}-NTA column chromatography flow-through; lane 3, purified PcaA protein (48 kDa).

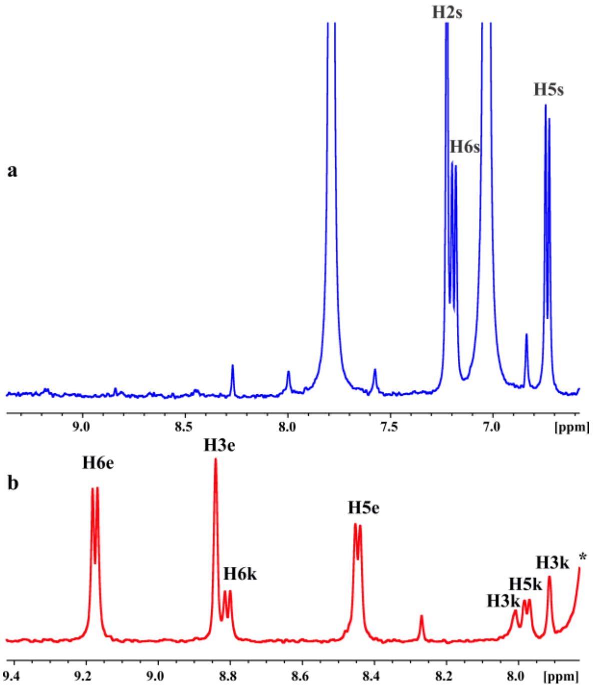

Supplementary Fig. S3. *In situ* monitoring of the biotransformation of PCA by PcaA, inside the NMR tube ^{1}H NMR spectra **(a)** 0 hours and **(b)** 24 hours after the addition of substrate.

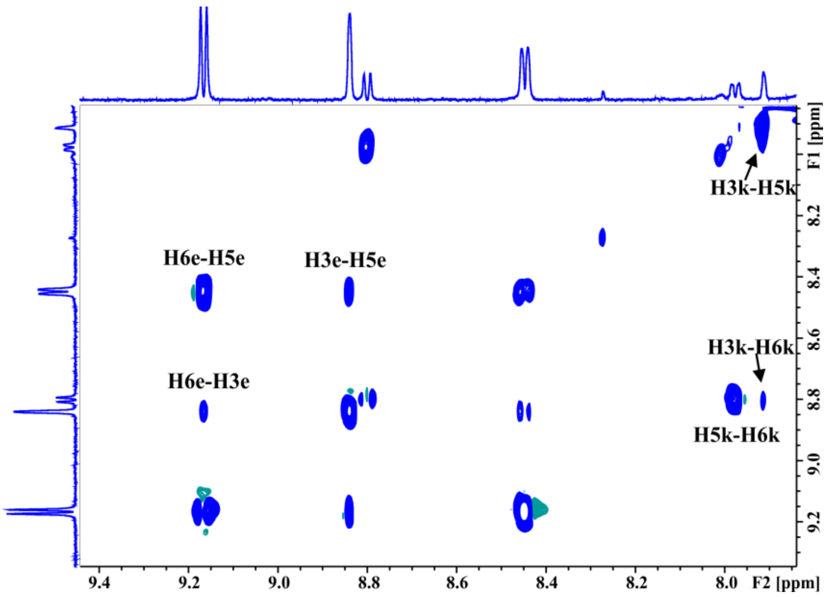

Supplementary Fig. S4. 2D TOCSY NMR spectrum of keto-enol forms of 4-carboxy-2-hydroxymuconate-6-semialdehyde reaction product (number of scans = 64, mixing time = 0.06s, total experimental time = 4h 40min).

The keto/enol equilibrium of the CHMS product was described by the equilibrium constant K_{eq}

$$K_{eq} = [\text{enol}]/[\text{keto}] \quad (1)$$

which is directly related to the Gibbs energy ΔG°

$$\Delta G^\circ = -RT\ln K_{eq} \quad (2)$$

Once the equilibrium constant can be determined at several temperatures, then, according to the Van't Hoff equation

$$\ln K_{eq} = -\Delta H^\circ/RT + \Delta S^\circ/R \quad (3)$$

the ΔH° and ΔS° terms can be obtained by the plot of $\ln K_{eq}$ versus 1/T. The line represents the best fit to eqs (2) and (3) with a correlation coefficient of 0.9988.

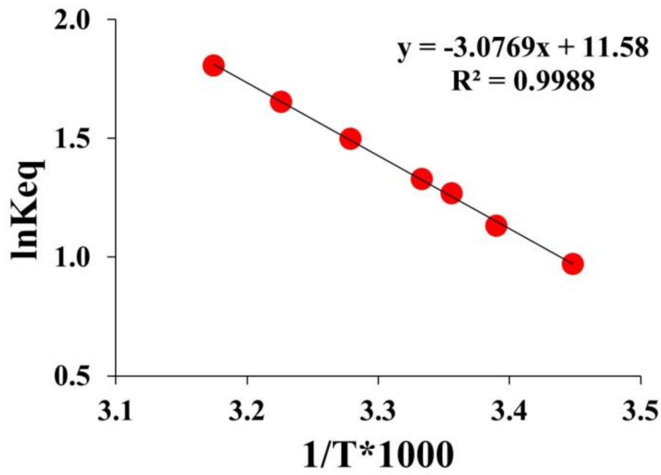

Supplementary Fig. S5. Representative Van't Hoff plot based on the enol/keto equilibrium NMR integration data of H3 protons in NMR buffer 90% H_2O (50mM Tris-HCl, 50mM NaCl, 0.25mM DTT, pH=8) with 10% DMSO-d_6. The solid line represents the best fit to eq (3).

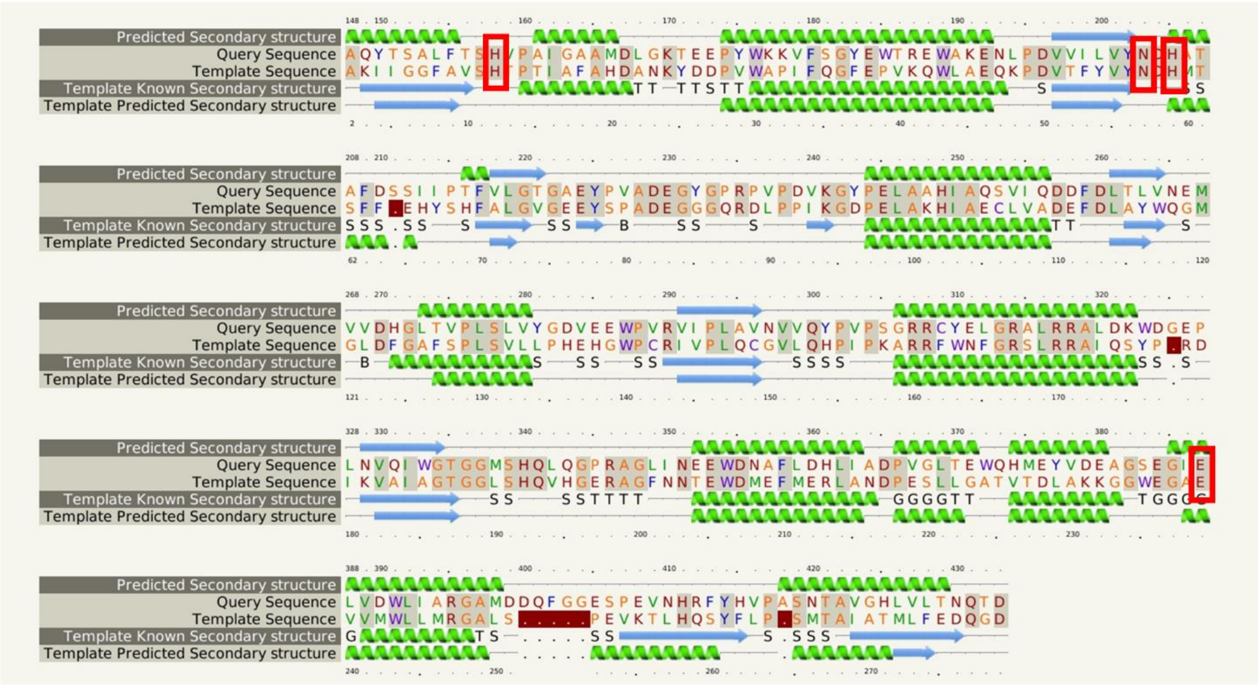

Supplementary Fig. S6: Sequence alignment of PcaA to the sequence of DesB. DesB is a type II extradiol dioxygenase catalyzing a ring opening reaction of gallate, derived from *Sphingobium* sp. SYK-6 [41]. Residues of DesB that coordinate to the non-heme Fe (II) are boxed (red). Relevant residues are conserved in PcaA and it is expected to share the same coordination geometry for Fe (II) (refer to Figure S7).

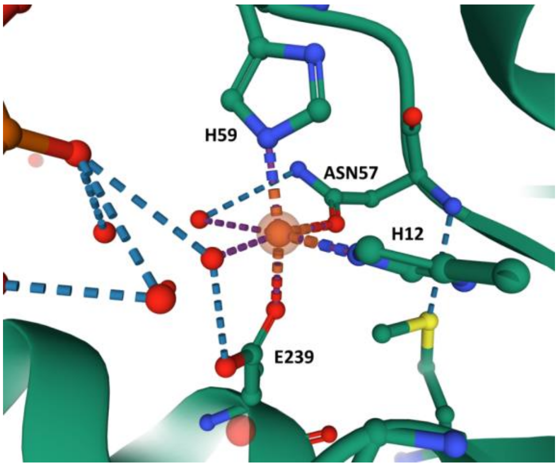

Supplementary Fig. S7: Coordination geometry of the non-heme Fe (II) in DesB (pdbid: 3WRB). H12, H59, N57 and E239 are conserved in PcaA to H35β, N80β, H82β and E264β, respectively.

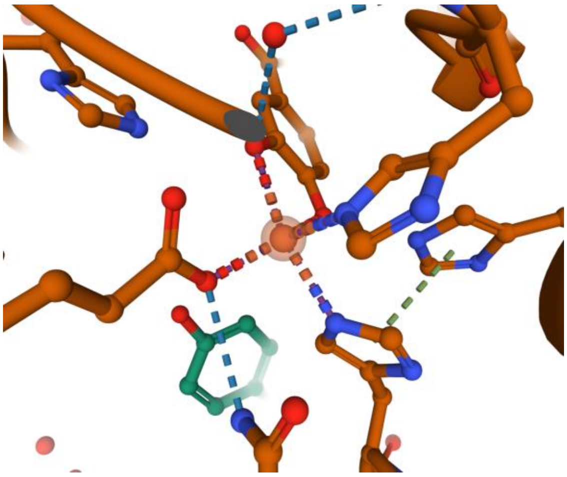

Supplementary Fig. S8: Coordination geometry of the non-heme Fe (II) in LigAB in complex with PCA (pdbid: 1B4U). H12, H61, N59 and E242 are conserved in PCA to H35β, H82β, N80β and E264β, respectively.

The pairwise alignment of the PcaA sequence with the sequences of known structures used for the modelling can be found in the embedded.pdf.

Supplementary Table S1: Oligonucleotides used in this study. Highlighted nucleotides indicate the restriction sites for *Nde*I (5'-CA/TATG-3') and *Xho*I (5'-C/TCGAG-3') that facilitated pET29c cloning.

Designation	Oligonucleotide sequence	Purpose	Size (bp)
$pca45diox_{for}$	5'-CCCGAAGGAGTATCATATGACACTCGAC-3'	pET29c cloning	1302
$pca45diox_{rev}$	5'-ATGCGGTCACTCGAGCTGGTTC-3'	pET29c cloning	
$pca45\alpha\beta_{for}$	5'-ACACCTCGGCACTATTCACC-3'	RT-qPCR	162
$pca45\alpha\beta_{rev}$	5'-CGTTGTACACCAGGATGACG-3'	RT-qPCR	
$pcareg_{for}$	5'-ACGCCTTGGCACAAATAGAC-3'	RT-qPCR	178
$pcareg_{rev}$	5'-TCGAGCCGTTCTACCTGAAT-3'	RT-qPCR	
$pcaRT1_{for}$	5'-AAT ACG TCC GAT CCC ATG AC-3'	RT-PCR	583
$pcaRT1_{rev}$	5'-TCA TGA GCT TGA GCT GGT TG-3'	RT-PCR	
$pcaRT2_{for}$	5'-CATCGACCTTCTCACCAAGG-3'	RT-PCR	450
$pcaRT2_{rev}$	5'-GTTGTCACCAGGCTGTAGCA-3'	RT-PCR	
$pcaRT3_{for}$	5'-GATAACGAGGAAGCCAAACG-3'	RT-PCR	362
$pcaRT3_{rev}$	5'-CGCCTGTACGATGACGTT C-3'	RT-PCR	
$pcaRT4_{for}$	5'-GTCGACTACATCCCGCAGAT-3'	RT-PCR	438
$pcaRT4_{rev}$	5'-CCGATCATCATGTCTCGGTA-3'	RT-PCR	
$pcaRT5_{for}$	5'-GAATGGCAGCACATGGAGTA-3'	RT-PCR	515
$pcaRT5_{rev}$	5'-ACGGAATCTCGACCTGCAC-3'	RT-PCR	
$pcaRT6_{for}$	5'-ACGGTATTGAGCTGCAGGAT-3'	RT-PCR	358
$pcaRT6_{rev}$	5'-CGTCCATCGATGACTTTCAC-3'	RT-PCR	
$pcaRT7_{for}$	5'-CACCTGCGTGAGAACATGG-3'	RT-PCR	470
$pcaRT7_{rev}$	5'-CTCGTCGGCATTCGTAAAG-3'	RT-PCR	
